# Temperature-Dependence of Weibel-Palade Body Exocytosis and Cell Surface Dispersal of von Willebrand Factor and Its Propolypeptide

**DOI:** 10.1371/journal.pone.0027314

**Published:** 2011-11-11

**Authors:** Lindsay Hewlett, Gregor Zupančič, Gregory Mashanov, Laura Knipe, David Ogden, Matthew J. Hannah, Tom Carter

**Affiliations:** 1 Division of Physical Biochemistry, Medical Research Councils National Institute for Medical Research, London, United Kingdom; 2 Department of Biology, University of Ljubljana, Ljubljana, Slovenia; 3 Brain Physiology Laboratory, Université René Descartes, Paris, France; Leiden University Medical Center, The Netherlands

## Abstract

**Background:**

Weibel-Palade bodies (WPB) are endothelial cell (EC) specific secretory organelles containing Von Willebrand factor (VWF). The temperature-dependence of Ca^2+^-driven WPB exocytosis is not known, although indirect evidence suggests that WPB exocytosis may occur at very low temperatures. Here we quantitatively analyse the temperature-dependence of Ca^2+^-driven WPB exocytosis and release of secreted VWF from the cell surface of ECs using fluorescence microscopy of cultured human ECs containing fluorescent WPBs.

**Principal Findings:**

Ca^2+^-driven WPB exocytosis occurred at all temperatures studied (7–37°C). The kinetics and extent of WPB exocytosis were strongly temperature-dependent: Delays in exocytosis increased from 0.92 s at 37°C to 134.2 s at 7°C, the maximum rate of WPB fusion decreased from 10.0±2.2 s^−1^ (37°C) to 0.80±0.14 s^−1^ (7°C) and the fractional extent of degranulation of WPBs in each cell from 67±3% (37°C) to 3.6±1.3% (7°C). A discrepancy was found between the reduction in Ca^2+^-driven VWF secretion and WPB exocytosis at reduced temperature; at 17°C VWF secretion was reduced by 95% but WPB exocytosis by 75–80%. This discrepancy arises because VWF dispersal from sites of WPB exocytosis is largely prevented at low temperature. In contrast VWF-propolypeptide (proregion) dispersal from WPBs, although slowed, was complete within 60–120 s. Novel antibodies to the cleaved and processed proregion were characterised and used to show that secreted proregion more accurately reports the secretion of WPBs at sub-physiological temperatures than assay of VWF itself.

**Conclusions:**

We report the first quantitative analysis of the temperature-dependence of WPB exocytosis. We provide evidence; by comparison of biochemical data for VWF or proregion secretion with direct analysis of WPB exocytosis at reduced temperature, that proregion is a more reliable marker for WPB exocytosis at reduced temperature, where VWF-EC adhesion is increased.

## Introduction

Weibel-Palade bodies (WPBs) are the principle regulated secretory organelle of endothelial cells (ECs) and contain the haemostatic protein von Willebrand factor (VWF) and the VWF-propolypeptide (proregion) in a 1∶1 stoichiometry [1]. Other proteins, most notably the integral membrane protein P-selectin, are also stored within WPBs [2], [3], [4]. VWF is synthesized as a pre-pro-protein [1]. The signal peptide (pre) that directs the nascent VWF polypeptide into the endoplasmic reticulum (ER) is cleaved co-translationally giving rise to proVWF within the lumen of the secretory pathway and the proregion is subsequently cleaved from the main VWF peptide in the trans Golgi network (TGN) and WPB [5]. Under the low pH and high Ca^2+^ conditions of the TGN (and subsequently the WPB itself) proregion remains non-covalently associated with mature disulphide linked VWF multimers to form ordered helical tubules of proregion-VWF [6], [7]. The proregion-VWF tubules give rise to the extended rod-like morphology of the WPB [7] and are now known to help facilitate the retention and concentration of P-selectin within the WPB membrane [8]. The regulated exocytosis of high molecular weight VWF multimers and P-selectin from WPBs plays an important role in facilitating platelet capture and regulating the initial attachment of neutrophils to the vessel wall under flow conditions at sites of vascular activation or injury [1].

To date, there has been no direct analysis of the temperature-dependence of WPB exocytosis from ECs. Biochemical assays of secretagogue-evoked VWF secretion from ECs suggest that WPB exocytosis is blocked at 18–20°C [9]. However, indirect evidence indicates that WPB exocytosis may occur at much lower temperatures, such as those used for hypothermic organ preservation both in animal models and in humans [10].

Here we report a direct and quantitative analysis of the temperature-dependence of Ca^2+^-driven WPB exocytosis and of the kinetics of dispersal of secreted fluorescent fusion proteins of VWF and proregion [11] from individual WPBs of human ECs. A discrepancy was found between optical data of WPB exocytosis observed directly and biochemical data of secreted VWF at sub-physiological temperatures. This is due to the retention of VWF at the cell surface at low temperature. Novel antibodies specific for cleaved and processed proregion are described and used to show that secretion of the soluble proregion is a more reliable marker for WPB exocytosis than secreted VWF under conditions of low temperature.

## Results

### Temperature-dependence of WPB exocytosis

**Figure 1:**
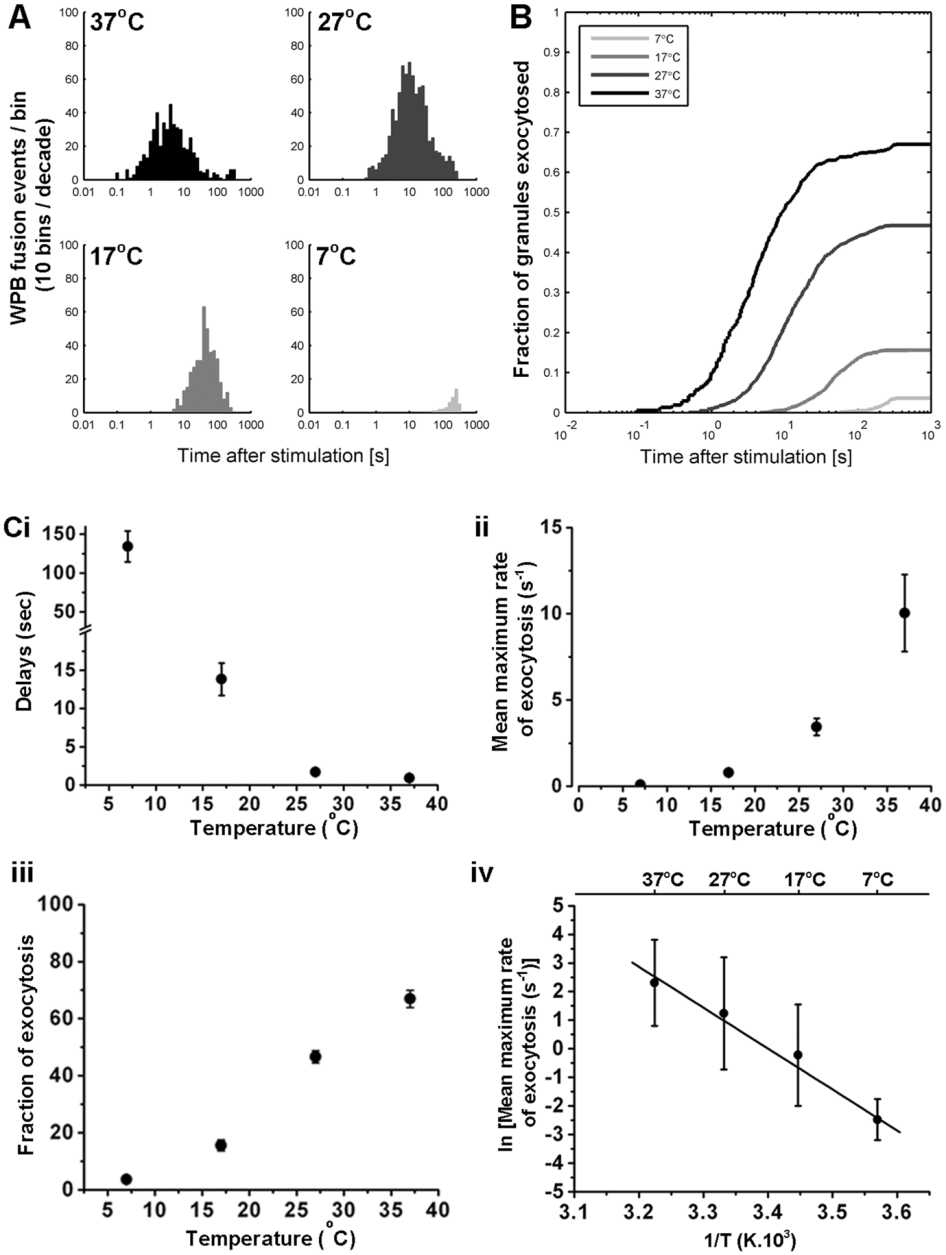
Temperature-dependence of Ca^2+^-driven WPB exocytosis. Panel A. Histogram of elapsed times from application of Ionomycin to WPB exocytosis. Log-binned time axis. Ionomycin (1 µM) evoked WPB exocytosis was determined with TIRF EMCCD imaging in proregion-EGFP expressing HUVEC at 4 temperatures: 37°C (n = 507 WPBs, 41 cells), at 27°C (n = 802 WPBs in 18 cells), at 17°C (n = 414 WPBs, 17 cells), and at 7°C (n = 43 WPBs, 10 cells). Panel B shows the same data normalised as the fraction of WPB in each cell that have undergone exocytosis in the time interval after stimulation, plotted on a log scale. Data are shown for the temperatures 37°C, 24°C, 17°C and 7°C. At lower temperature the latency to the first event in each cell is increased and the fraction of WPB undergoing exocytosis in each cell is reduced. Panels Ci–iii show the mean delays between ionomycin application and the first detected fusion event at each temperature, the mean of maximal rates of exocytosis as a fraction of the total in each cell, and the final fraction of WPB that underwent exocytosis (expressed as the percent of the fluorescent WPBs that fused with the plasma membrane) respectively. Panel Civ shows an Arrhenius plot for the maximal rate of WPB exocytosis fitted with a linear regression (solid black line, R = −0.99, p = 0.005, slope −59.9 kJ/mol).

Proregion-EGFP expression in HUVEC is localised in WPBs and permits a direct and time-resolved analysis of WPB exocytosis in living cells [11], [12]. Stimulation with the calcium ionophore, ionomycin was used to bypass the potentially confounding temperature-dependent effects on hormone-receptor activity and signal transduction cascades, allowing direct analysis of the temperature dependence of Ca^2+^-driven WPB exocytosis [12]. Since exocytosis of each WPB can be observed as a separate event, histograms were constructed of the times from ionomycin addition to exocytosis at different temperatures. These latency distributions for ionomycin-evoked WPB exocytosis at 37, 27, 17 and 7°C are summarised in Figure 1A as log-binned histograms of number of exocytotic events with time. The data are plotted in cumulative form in Figure 1B, as the fraction of WPB fused in each cell against time (log time scale). The data show long delays (after adding ionomycin) before secretion of individual WPBs and a strong dependence on temperature. Further, the fraction of WPBs that exocytose also shows a strong dependence on temperature. The data are analysed in Figure 1C. The mean delays between ionomycin application and the first detected fusion event at each temperature were 0.92±0.13 s (37°C; n = 41 cells), 1.72±0.47 s (27°C; n = 18 cells), 13.84±2.11 s (17°C; n = 17 cells) and 134.17±19.68 s (7°C; n = 10 cells) (Figure 1Ci). The mean maximal rates of exocytosis were 10.04±2.23 s^−1^ (37°C), 3.44±0.48 s^−1^ (27°C), 0.80±0.14 s^−1^ (17°C) and 0.08±0.04 s^−1^ (7°C) (Figure 1Cii), and the fractional extent of degranulation (during ∼300 s of stimulation) were 66.9±3.0% (37°C), 46.6±2.1% (27°C), 15.6±1.9% (17°C) and 3.6±1.3% (7°C) (Figure 1Ciii). An Arrhenius plot for the maximal rate of WPB exocytosis (Figure 1Civ) yielded a high activation energy of ∼60 kJ/mol.

### Temperature-dependence of VWF release into the medium

**Figure 2:**
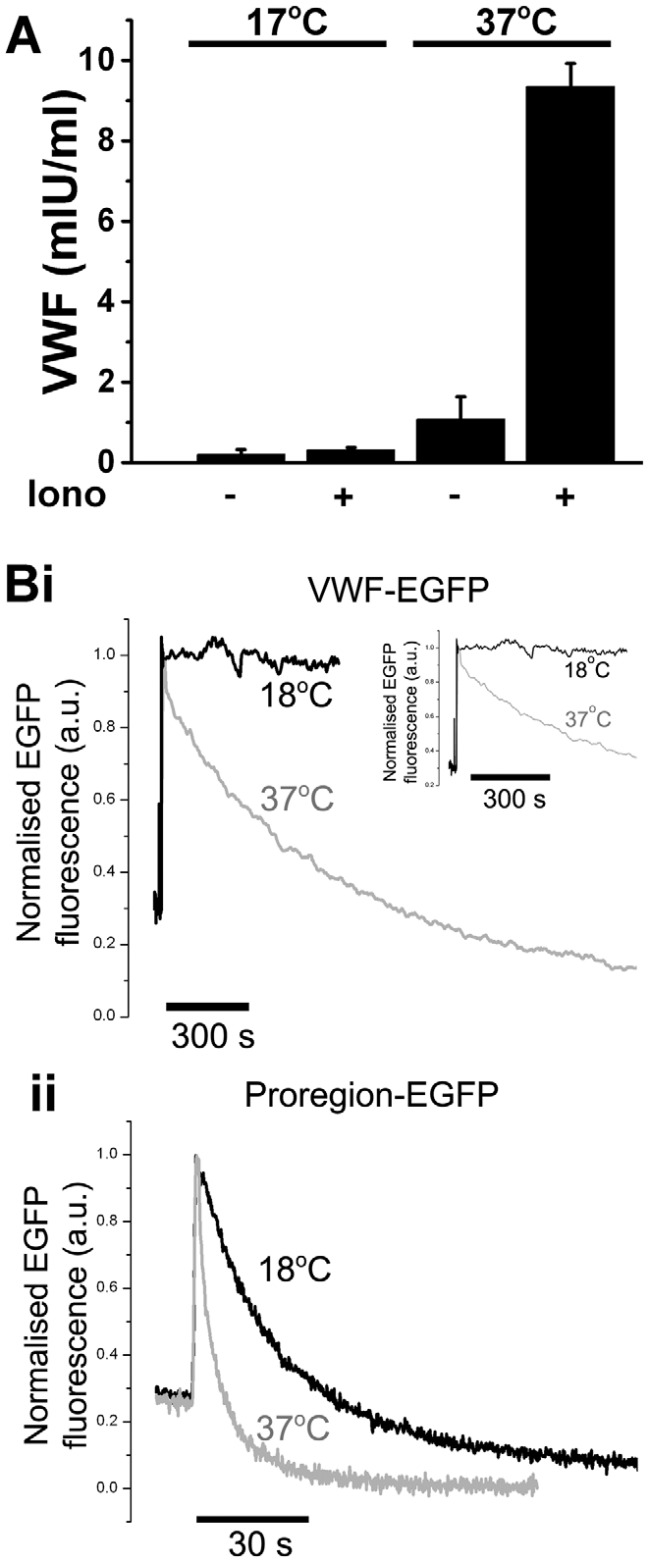
Effect of temperature on VWF secretion into the medium and dispersal of VWF-EGFP or proregion-EGFP from sites of WPB exocytosis. Panel A: shows ionomycin-evoked secretion of VWF at 17°C and 37°C. Data from one experiment carried out in triplicate and representative of 4 separate experiments. Cells were incubated in the absence (−) or presence (+) of ionomycin (1 µM) and secreted VWF assayed by ELISA. Panel B shows representative time-courses for the changes in fluorescence of VWF-EGFP (Bi) and proregion-EGFP (Bii) in individual WPBs undergoing exocytosis in HUVEC at 18°C (black traces) or 37°C (grey traces). Data at each temperature are aligned to the initial abrupt increase in EGFP fluorescence due to fusion pore formation [12] and are shown normalised to the peak increase in EGFP fluorescence upon WPB fusion. Fluorescence was measured in images acquired at 5 frames/sec. At times longer than 120 s imaging rate was reduced to 0.2 frames/sec. Excitation light was attenuated by a 50% neutral density filter. Inset in Bi is the initial time-course for VWF-EGFP dispersal shown on an expanded time scale.

**Figure 3:**
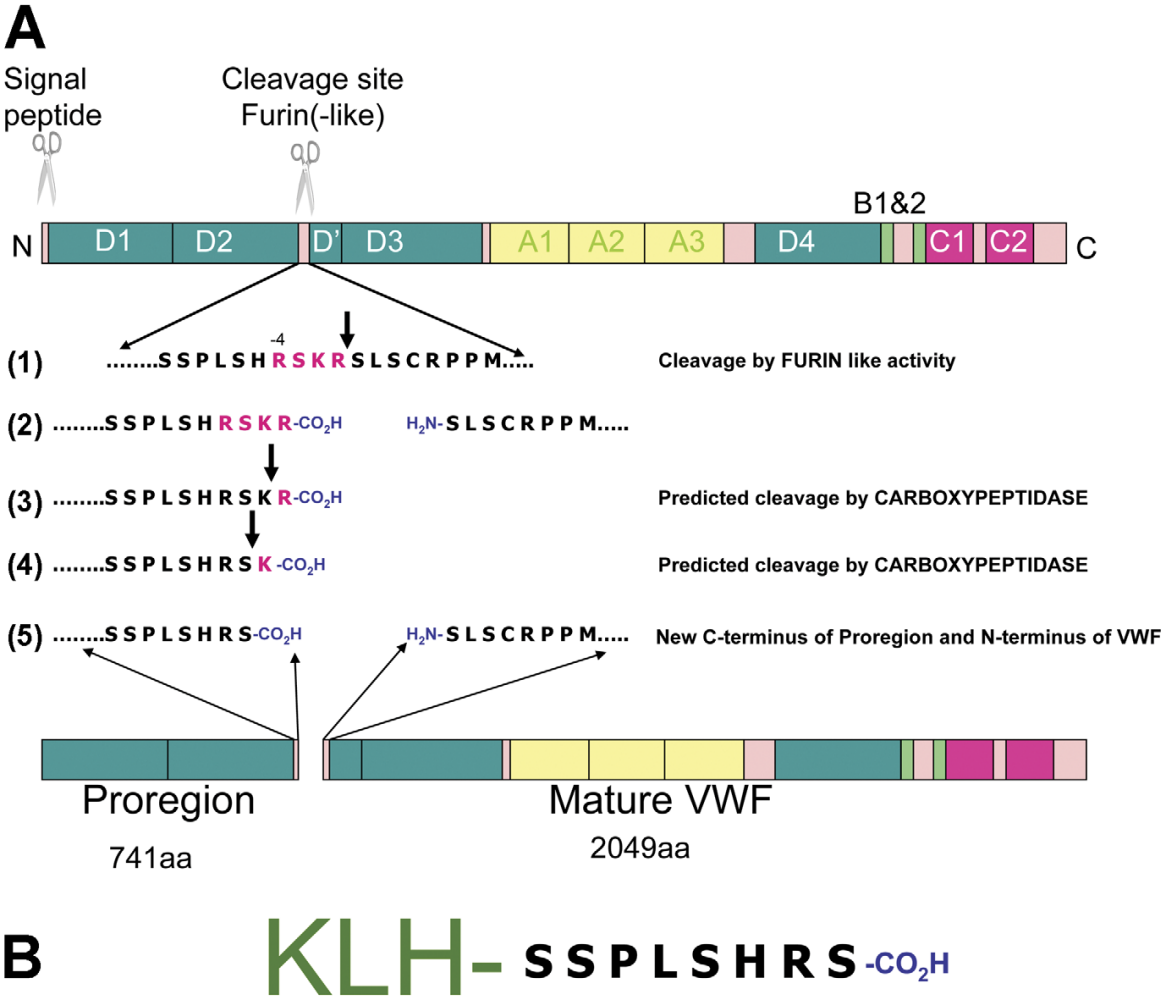
Strategy for making the proregion specific antibodies. VWF is synthesized as a pre-proprotein comprising an N-terminal signal peptide (pre-), and several distinct repeating structural domains (termed A,B,C & D) arranged as D1-D2-D′-D3-A1-A2-A3-D4-B1-B2-B3-C1-C2-CK (see cartoon). During translation the signal peptide is removed to yield proVWF which then undergoes disulphide-linked dimerisation to produce proVWF dimers [1] (not shown). The proregion domains (D1–D2) are cleaved from the main peptide in the TGN by a furin-like activity. Line (1) shows the amino acid sequence in the region that includes the tetrabasic cleavage site (shown in purple) and the vertical arrow indicates the predicted site of scission by furin (in line 1). Line (2) shows the resulting C-terminal region of proregion and the N-terminal region of mature VWF. Lines (3 & 4) show the predicted trimming of the C-terminal arginine (3) and lysine (4) residues by carboxypeptidases that gives rise to the putative final C-terminal sequence of proregion (5). Panel B shows the KLH-coupled synthetic octapeptide used for immunization.

In line with previous reports [9] biochemical analysis suggested that stimulated secretion of VWF is essentially abolished at 17°C (Figure 2A). However, direct optical observation showed that fusion events at 17°C were still 22% of those observed at 37°C (Figure 1Ciii). Because VWF adheres strongly to the cell surface at sites of release dispersing slowly into solution [11] we tested whether the apparent discrepancy between biochemical and optical studies at 17°C might reflect a further slowing of the dispersal of VWF from the cell surface. To do this we directly imaged the kinetics of fluorescent VWF (VWF-EGFP) dispersal from individual sites of exocytosis (Figure 2B black traces). For comparison we also analysed the kinetics of proregion-EGFP dispersal. The half-time for dispersal of VWF-EGFP at 37°C was 342±126.9 s (±SD, n = 39 WPB, n = 7 cells). The dispersal of VWF from sites of exocytosis was almost completely prevented at 18°C (black traces), showing no apparent dispersal over several minutes of observation. In contrast, the mean half time for dispersal of proregion-EGFP at 37°C was 2.56±1.78 s (n = 36 WPBs, n = 7 cells), while at 18°C it was 15.6±1.6 sec (n = 18 WPBs, n = 6 cells) (Figure 2C). Thus, proregion-EGFP dispersal, although slowed, was still rapid enough at 18°C to be complete within 1–2 minutes of fusion. The relatively rapid dispersal of proregion-EGFP at reduced temperature suggested that this soluble WPB component would represent a more reliable biochemical marker for WPB exocytosis under conditions of reduced temperature. To formally address this we developed assays specific for proregion using novel antibodies that were designed to recognise cleaved proregion, the product of proVWF processing that is found only in the WPB, and not biosynthetic proVWF or VWF that are present in other parts of the secretory pathway (e.g. ER and Golgi). The strategy for making proregion specific antibodies is summarized in Figure 3.

### Characterisation of anti-proregion specific antibodies

**Figure 4:**
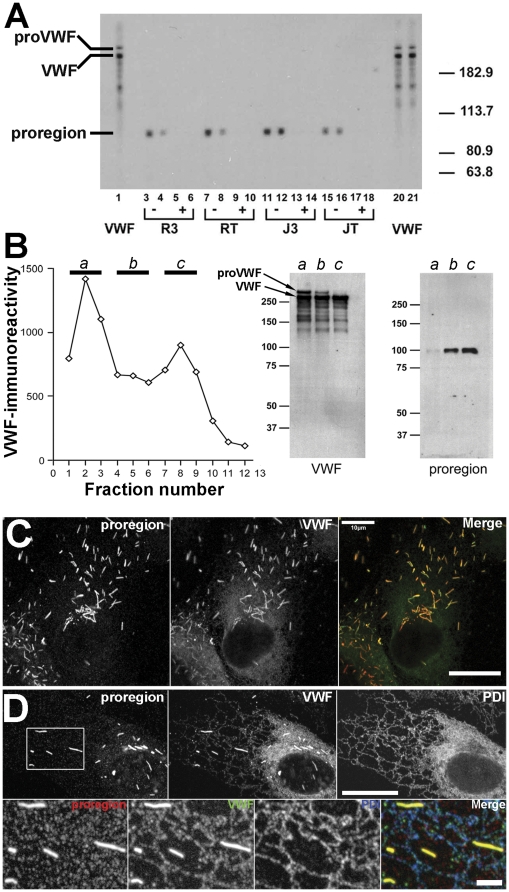
Characterisation of rabbit polyclonal anti-proregion antibodies. Panel A shows a representative immunoblot of a HUVEC lysate probed with rabbit antibodies specific for proregion. VWF = rabbit polyclonal anti-VWF (Dako). Robbie (R) and Justin (J) represent two different rabbits immunized with an octa-peptide (coupled to KLH) corresponding to the predicted carboxy-terminus of proregion after cleavage from proVWF. R3 & J3 represent the third bleeds, RT & JT represent the terminal bleeds. Plus and minus refer to inclusion of the peptide used for immunization (not coupled to KHL, +) or absence of peptide (−) during the incubations. Duplicate lanes were probed with each antibody-peptide combination. Binding of rabbit antibodies to the blot was revealed using an HRP-coupled secondary antibody followed by ECL. Panel B shows VWF-immunoreactivity (measured by ELISA) in fractions from a Percoll™ gradient fractionation of HUVEC PNS. Fraction density increases from 1 to 12. Right panel shows immunoblots of gradient fractions (a,b and c) pooled as indicated and probed with anti-VWF or anti-proregion (RT) antibodies. Positions of proVWF and mature VWF are indicated. Panel C shows the immunofluorescence localisation of proregion (RT; left panel and red in colour merge) and VWF (sheep anti-VWF; middle panel and green in colour merge) immunoreactivities in HUVEC. Images for each antigen were acquired sequentially as single, confocal, optical sections through a representative cell. Scale bar is 10 µm. Panel D shows the immunofluorescence localisation of proregion and VWF as above and PDI (mouse monoclonal; right panel) in HUVEC, scale bar is 10 µm. The region indicated by the white box in the left panel is shown on expanded scale below with proregion; red, VWF, green, PDI blue in colour merge image. (N.B. The brightness and contrast for proregion have been “pushed” beyond saturation in the bottom panel to demonstrate the absence of reticular (ER) staining for proregion. This has accentuated the granular background staining (not displaceable by peptide -data not shown) Scale bar is 2 µm.

**Figure 5:**
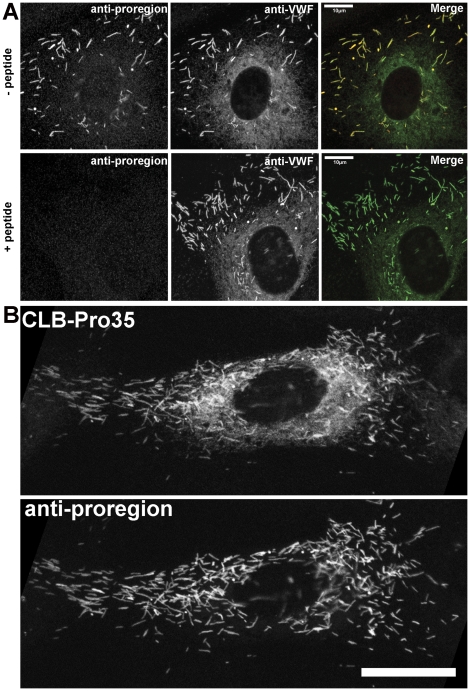
Anti-proregion antiserum specifically recognizes WPBs and not other biosynthetic compartments. Panel A shows double immunofluorescence images of HUVEC stained for proregion (RT; red in colour merge) and VWF (sheep-anti-VWF; green in colour merge) in the absence (top panels) or presence (lower panels) of a large molar excess of the synthetic octapeptide used to raise the proregion antiserum. Panel B shows a double immunofluorescence image of a single HUVEC stained with the mouse monoclonal antibody CLB-Pro35 that was raised to a non-furin cleavable form of proVWF [16] (top panel) and anti-proregion antiserum (RT) (lower panel). Both antibodies recognize WPBs, but CLB-Pro35 also detects a diffuse staining that represents proVWF within the ER. Scale bar is 10 µm.

Proregion-specific antisera derived from the third bleeds (R3 and J3) and terminal bleeds (RT and JT) of two rabbits, Robbie (R) and Justin (J), were used to probe HUVEC lysates by immunoblotting (Figure 4A). Only one immunoreactive band of the apparent molecular weight (100 kDa) predicted for proregion was observed (Figure 4, duplicate lanes 3&4, 7&8, 11&12, 15&16). This immunoreactivity was not seen if the antisera were pre-incubated with the peptide used for immunization (Figure 4, lanes 5&6, 9&10, 13&14, 17&18). Probing the same preparative blot with a commercial antibody recognising VWF (lanes 1, 20&21) did not recognise the proregion band but picks up two higher molecular weight bands that we know from previous studies [13] represent mature VWF and proVWF (Figure 4A) (as well as some lower molecular weight “smear” that presumably represents proteolytic cleavage products of VWF). The proregion antisera did not recognise a protein corresponding to the molecular weight of proVWF even on an overexposure of the blot (shown in Figure S1). Because the cleaved and processed proregion should be enriched in WPBs and absent from early biosynthetic compartments (ER) we next undertook subcellular fractionation of post nuclear supernatants of HUVEC to separate the early biosynthetic compartments from mature WPBs using density gradient centrifugation [14]. Figure 4B (left) shows a representative example of fractions from such a density gradient probed for VWF immunoreactivity. Previous studies [15] have shown that the peak in light fractions (here fractions 1–3) correspond to VWF within the ER and Golgi while the peak at dense fractions (7–9) corresponds primarily to VWF in mature WPBs, with some overlap between these two pools in the intermediate density fractions. Levels of VWF immunoreactivity in the dense fractions are reduced in cells that have been exposed to WPB secretagogue prior to fractionation while VWF in the light fractions are unaffected by secretagoge, but reduced after inhibition of protein synthesis with cycloheximide (LH and MJH personal communication). Material from light fractions (a; fractions 1–3), medium density fractions (b; fractions 4–6) and the dense fractions (c; fractions 7–9) were pooled and probed by Immunoblot using anti-VWF or anti-proregion antibodies (Figure 4B right). VWF was detected in all fractions while the higher molecular weight band corresponding to proVWF was seen principally in the light fractions, to a lesser extent in the intermediate density fractions and was absent from the dense fractions. As expected proregion immunoreactivity was enriched in the dense fractions and largely absent from the light fractions. Immunofluorescence of PFA-fixed HUVEC using the proregion antibodies illuminated WPBs, as did antibodies to mature VWF (Figure 4C). Proregion staining was completely displaced by pre-incubation with the peptide to which the antisera was raised (Figure 5A). However, antibodies to VWF also gave rise to a diffuse/reticular staining throughout the cell that is not seen with the anti-proregion antibodies described here, but which was also seen with the monoclonal anti-proregion antibody, CLB-Pro35 (this antibody was raised to an uncleavable mutant of proVWF (pro-vWFgly76 [16] (Figure 5B) and consequently would be expected to recognise its proregion epitope both after cleavage and also while still part of proVWF). The diffuse/reticular staining co-localised with the ER luminal protein disulphide isomerise (PDI) (Figure 4D) consistent with it representing biosynthetic proVWF in the early part of the secretory pathway, before its cleavage into mature VWF and proregion.

### Proregion is a more reliable marker of WPB exocytosis at reduced temperature

**Figure 6:**
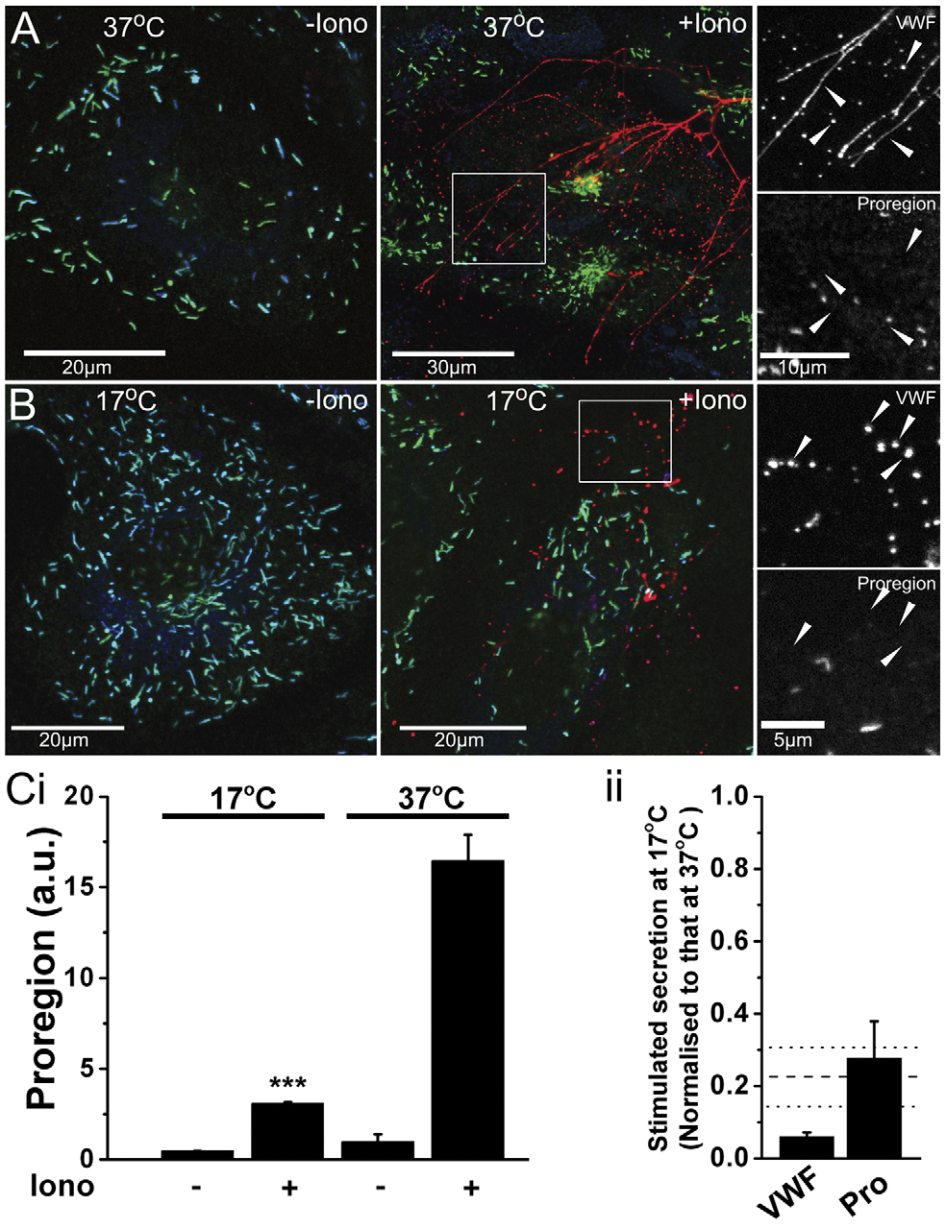
Proregion secretion more reliably reports WPB exocytosis at reduced temperature. Panel A shows colour merged images of extracellular VWF (red; rabbit anti-human VWF, Dako Ltd, Ely, UK), intracellular VWF (green; sheep anti-human VWF, Serotec, Kidlington, UK) and proregion (blue; affinity purified chicken polyclonal antibody specific to proregion described above) immunoreactivity on control (−Iono) and stimulated (+Iono; 1 µM) cells at the temperatures indicated. Grey scale panels on the right show regions indicated by the white boxes on expanded scales. Large arrowheads in the grey scale images indicate the position of extracellular VWF and the absence of associated proregion. Images for each antigen were acquired sequentially as single, confocal, optical sections. (Images for the same antigen were acquired using the same confocal settings). Panel Ci shows ionomycin-evoked secretion of proregion at 17°C and 37°C. Data shown are from one experiment carried out in triplicate and are representative of 4 separate experiments. *** P<0.0001. Panel Cii summarises the reduction in stimulated secretion of VWF or proregion (Pro) at 17°C in the 4 experiments. Data are normalised to secretion at 37°C. Horizontal dashed line indicates the mean extent of WPB exocytosis at 17°C (normalised to that at 37°C; horizontal dotted lines indicate SE of the mean extent of WPB exocytosis).

Consistent with live cell imaging studies (Figure 2), triple immuno-staining for extracellular VWF-immunoreactivity (red), intracellular VWF-immunoreactivity (blue) and proregion-immunoreactivity (green) in control and ionomycin stimulated cells at 37°C (Figure 6, see also Figure S2) or 17°C (Figure 6B see also Figure S2) confirmed that proregion does not remain associated with extracellular VWF after WPB exocytosis at either temperature. In addition it was noted that at 17°C VWF failed to form long strings on the cell surface, characteristic of its appearance at 37°C, but instead comprised globular deposits (Figure 6B). Using the proregion specific sandwich ELISA described here (and used previously 12) we measured secretion of proregion at 37°C or 17°C (Figure 6Ci). In contrast to VWF (Figure 2A), a significant increase in soluble proregion was observed in media at 17°C. The levels of soluble VWF and proregion in media at 17°C, normalised to that at 37°C, are compared in Figure 6Cii. The horizontal dashed and dotted lines show the mean and ±SD, respectively, for the extent of WPB degranulation at 17°C, normalised to that at 37°C.

## Discussion

Direct analysis of the exocytosis of fluorescent WPBs in living ECs revealed that the delays, rates and fractional extent of WPB exocytosis were all strongly temperature sensitive, with particularly large changes for the delays between Ca^2+^-stimulation and fusion and maximum rates occurring between 17°C and 7°C. The latter changes may reflect in part a lipid membrane phase transition that is reported to occur in biological membranes at between ∼15°C and 5°C [17], and which leads to reduced membrane fusion. An Arrhenius plot for the maximal rate of WPB exocytosis (Figure 1Civ) yielded an activation energy of ∼60 kJ/mol supporting the requirement of ATP-dependent processes in Ca^2+^-driven WPB exocytosis [18].

WPB exocytosis is most commonly studied by analysing soluble VWF accumulated in the external medium. Based on these assays the conclusion that WPB exocytosis is blocked at 17°C arose from the observation that the stimulated appearance of soluble VWF in media of cultured ECs is largely abolished at this temperature [9]. We confirmed this result showing a 95% inhibition, but also showed by direct observation of exocytosis of fluorescent WPBs that this reduction in VWF appearance in the medium at low temperature considerably overestimates the effect of temperature on exocytosis. Based on imaging of VWF-EGFP following stimulation we show that this discrepancy can be explained by a strong temperature dependence of VWF dispersal from the cell surface. VWF from WPBs comprises high molecular weight multimers [1] with each polymer containing many sites for interaction with cell surface components. The polyvalent nature of VWF facilitates its adhesion to the cell surface to support platelet recruitment to the vessel wall [5] and may contribute to its slow (compared to other soluble secreted molecules released from the WPB [11], [19]) dispersal from the EC surface. VWF dispersal will depend on the number, properties and frequency of its interactions with cell surface ligands, its susceptibility to fluid shear forces and, potentially, to proteolytic processing to smaller fragments by circulating or EC surface associated proteases such as ADAMTS13. Many of these processes may be temperature sensitive. A striking feature seen here was the failure of secreted VWF to form long cell surface strings following its secretion at 17°C (Figure 6B); instead VWF formed predominantly globular deposits. This failure of VWF to unfurl at 17°C, coupled with the likelihood of reduced thermal motions may interfere with the processes that underlie VWF dispersal resulting in the prolonged retention of this protein on the cell surface.

The discrepancy between biochemical measurements of soluble secreted VWF and underlying WPB exocytosis at sub-physiological temperatures suggests the need for a more reliable biochemical marker of WPB exocytosis. Proregion and VWF are stored in equimolar amounts within WPBs [1] and the rapid dispersal of proregion-EGFP into solution even at reduced temperatures suggested that this molecule might be a better marker than VWF itself. To this end we raised and characterised antibodies to the theoretical carboxy terminus of proregion produced after furin cleavage and subsequent carboxypeptidase trimming (Figure 3). This epitope is unique to the WPB and should be absent from biosynthetic compartments within the cell. The antisera produced recognised only a single band of the predicted size for proregion (approximately 100 kDa) in immunoblots of HUVEC extracts, a single band of the same size enriched in dense fractions from percoll™ gradients of post-nuclear supernatants of HUVEC enriched in WPBs [15], [20], and unlike anti-VWF antibodies, decorated only WPBs and no other part of the biosynthetic pathway. Consistent with the rapid dispersal of proregion-EGFP from sites of WPB exocytosis we found no proregion-immunoreactivity associated with extracellular VWF at the cell surface after stimulation at either 37°C or 17°C (Figure 6B). Instead, and in contrast to VWF, we found a significant increase in proregion in media of cells stimulated at 17°C (Figure 6Ci), the magnitude of which was consistent with the extent of WPB exocytosis observed (Figure 6Cii). Together these data suggest that proregion is a much more reliable marker for WPB exocytosis than VWF, especially under conditions where VWF-EC adhesion is increased. In fact, because secreted proregion is cleared rapidly from the blood while VWF remains in the circulation at relatively high levels [16], proregion-specific assays such as the one described here represent a more precise way of assessing acute changes in vascular WPB secretory activity (compared with VWF assays) even under physiological conditions.

Our observation that Ca-driven WPB exocytosis can still occur at temperatures close to those used for hypothermic organ preservation are consistent with previous suggestions that release of WPB components may occur under very low temperature conditions [10]. Whether and to what extent the release of WPB components under such conditions impact on the inflammatory status of EC, and in turn tissue function, during procedures such as hypothermic organ transplant requires further study.

## Methods and Materials

### Tissue Culture, Nucleofection, Reagents, Immunocytochemistry and ELISA measurements

Cryopreserved, pooled, primary human umbilical vein endothelial cells (HUVEC; PromoCell, Heidelberg, Germany), were grown and Nucleofected as described previously [8], [11], [19], [21]. VWF-EGFP and proregion-EGFP were made as described [11]. A monoclonal antibody to protein disulphide isomerise (PDI, clone 1D3) was from Enzo Life Sciences (Exeter, UK), rabbit anti-human VWF was from Dako Ltd, (Ely, UK), sheep anti-human VWF was from Serotec (Kidlington, UK), and a monoclonal antibody to the VWF pro-peptide (CLB-Pro35) was as previously described [16]. Fluorophore and peroxidase coupled secondary antibodies were from Jackson Immunoresearch Laboratories and Percoll™ was purchased from GE Healthcare (Hertfordshire, UK). Unless otherwise stated all other reagents were from Sigma-Aldrich, (Poole Dorset, UK). Immunocytochemistry and ELISA for soluble secreted VWF were previously described [19]. Media and lysates were also assayed for proregion using a sandwich ELISA. ELISA plates were coated with 1.15 µg/ml monoclonal CLB-Pro35 while detection used an affinity-purified rabbit polyclonal antibody specific to the putative C-terminus of proregion (described above) followed by a horseradish peroxidase (HRP) conjugated anti-rabbit antibody. Blocking, washing and peroxidase measurement were as described for the VWF ELISA (12). Proregion concentrations were calculated from a standard curve made up of serial dilutions of normal human serum and expressed as arbitrary units.

### Total internal reflection fluorescence (TIRF) imaging of WPB exocytosis

Nucleofected HUVEC were plated onto poly-d-lysine coated 35 mm glass bottom dishes (MatTek Corporation, Ashland, MA) or 25 mm glass coverslips and cultured as described [8], [11]. For TIRF experiments 25 mm coverslips were transferred into Hank's balanced salt solution containing 20 mM HEPES (pH 7.4) and mounted into stainless steel imaging chambers sealed by a silicone O-ring as described [21]. The top of the chamber was open allowing access for secretagogue addition. The microscope was enclosed in an incubator (Solent Scientific, Segensworth, UK) allowing experiments at 37°C or 27°C respectively and also incorporated Peltier cooling elements to cool the objective lens and imaging chamber [21] allowing studies at 7°C or 17°C. The top of the cooling chamber incorporated a small removable lid allowing direct access to the chamber for secretagogue (pre-cooled to the appropriate temperature) addition. For experiments at 7°C or 17°C, cells were placed in the cooling stage and maintained for ∼30 minutes to equilibrate to the cooling stage temperature. An objective based TIRFM system [21] was used and images acquired at 30 frames/sec as described [21], [22]. WPB fusion was determined from abrupt fluorescence and morphological changes in WPBs as previously described [12]. Epifluorescence measurements of EGFP dispersal from individual fusion sites were carried out as previously described [11], [19], [23]. Room temperature was maintained at 18°C by air conditioning unit and temperature monitored at the specimen chamber using a thermocouple and thermometer.

### Antibodies to proregion, immunoblotting and subcellular fractionation

Antisera were raised in rabbit, chicken and guinea-pig to a *keyhole limpet* hemocyanin (KLH)-coupled synthetic octapeptide representing the theoretical carboxy terminus of the propolypeptide of proVWF (proregion) generated after furin cleavage and subsequent carboxypeptidase trimming (KLH-SSPLSHRS; see Figures 3 & S1). Their characterisation is illustrated here using the rabbit antisera. All experiments performed so far indicate that the chicken and guinea-pig antisera behave similarly (data not shown). Rabbit and chicken antibodies were affinity purified from heat inactivated serum or solvent extracted egg yolk, respectively, using the octapeptide used for immunization coupled to an Aminolink™ column (Thermofisher (Pierce products) Rockford, IL). For immunoblot analysis HUVEC lysates were prepared in SDS-PAGE sample buffer and proteins resolved on a preparative 6% acrylamide minigels. Electrophoresed proteins were transferred onto nitrocellulose and the membrane blocked. The blot was placed in a miniblotter™ apparatus (Immunetics, Web Scientific Ltd, Crewe. UK) (which separates the membrane into individual lanes) and blots probed with anti-VWF or putative rabbit anti-proregion antisera as described in the figure legends. Binding of rabbit antibodies to the blot was revealed using an HRP-coupled secondary antibody followed by ECL using Supersignal West Pico chemiluminescent Substrate (Pierce, Cramlington, UK), and exposure to film (GE Healthcare). Subcellular fractionation of confluent HUVEC was carried out by ultracentrifugation of post nuclear supernatants in self forming Percoll™ density gradients essentially as described previously [14]. For analysis by Western blot, pooled gradient fractions were centrifuged at 100,000 g for 2 hours at 4°C. Pelleted material was removed from the top of the percoll pellet and re-centrifuged at 100,000 g for 30 minutes at 4°C. The supernatant was removed and the pellet lysed in SDS-PAGE sample buffer. Proteins were resolved on a 6% polyacrylamide gel, transferred onto nitrocellulose and the membrane probed using anti-VWF and anti-proregion antibodies. Bound antibodies were visualised using HRP coupled secondaries and ECL as above.

### Analysis and statistics

Image analysis was carried out in ImageJ (http://rsbweb.nih.gov/ij/) or GMimPro, (custom-written, http://www.nimr.mrc.ac.uk/gmimpro/). Numbers of fluorescent WPBs were determined using the pointpicker plugin in ImageJ (http://bigwww.epfl.ch/thevenaz/pointpicker/). Data plotting, linear fitting and the statistical analysis (at 95% confidence limit) between population means, determined using a non-paired two way t-test, were carried out in Origin 7.5 (OriginLab Corporation, Northampton, MA, USA). Data are shown as mean±SE unless stated otherwise.

## Supporting Information

Figure S1 **Characterisation of anti-proregion antisera.** A longer exposure of the film shown in Fig. 4A, showing that anti-proregion antisera only recognize a single band at ∼100 KDa, the predicted size for proregion.
(TIF)

Figure S2 **WPB exocytosis at 37°C but not 17°C leads to the formation of long extracellular strings of VWF.** Colour merged images of extracellular VWF (red; rabbit anti-human VWF), intracellular VWF (green; sheep anti-human VWF) and proregion (blue; chicken polyclonal antibody specific to the putative C-terminus of human proregion [19] immunoreactivity on control (−Iono) and stimulated (+Iono) cells at the temperatures indicated. Grey scale panels on the right show regions indicated by the white boxes. Images for each antigen were acquired sequentially as single, confocal, optical sections. (Images for the same antigen were acquired using the same confocal settings). Scale bars are 50 µm.
(TIF)
