# Supplementary figures and images for: Temperature-Dependence of Weibel-Palade Body Exocytosis and Cell Surface Dispersal of von Willebrand Factor and Its Propolypeptide
Source: PLoS One. 2011 Nov 11;6(11):e27314. doi: 10.1371/journal.pone.0027314 (PMC3214045; doi:10.1371/journal.pone.0027314)

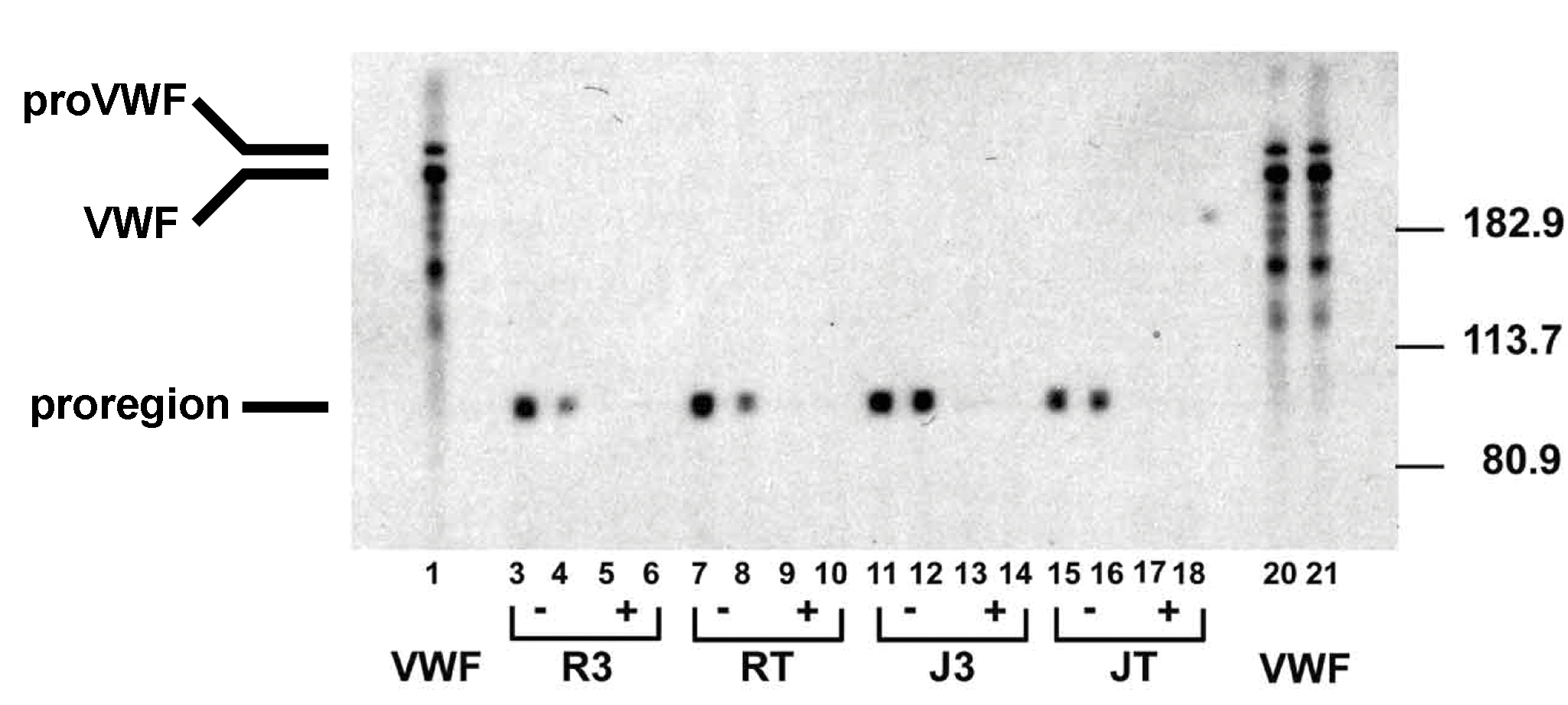

Supplement: Figure S1 — Characterisation of anti-proregion antisera. A longer exposure of the film shown in Fig. 4A, showing that anti-proregion antisera only recognize a single band at ∼100 KDa, the predicted size for proregion. (TIF) [file pone.0027314.s001.tif]

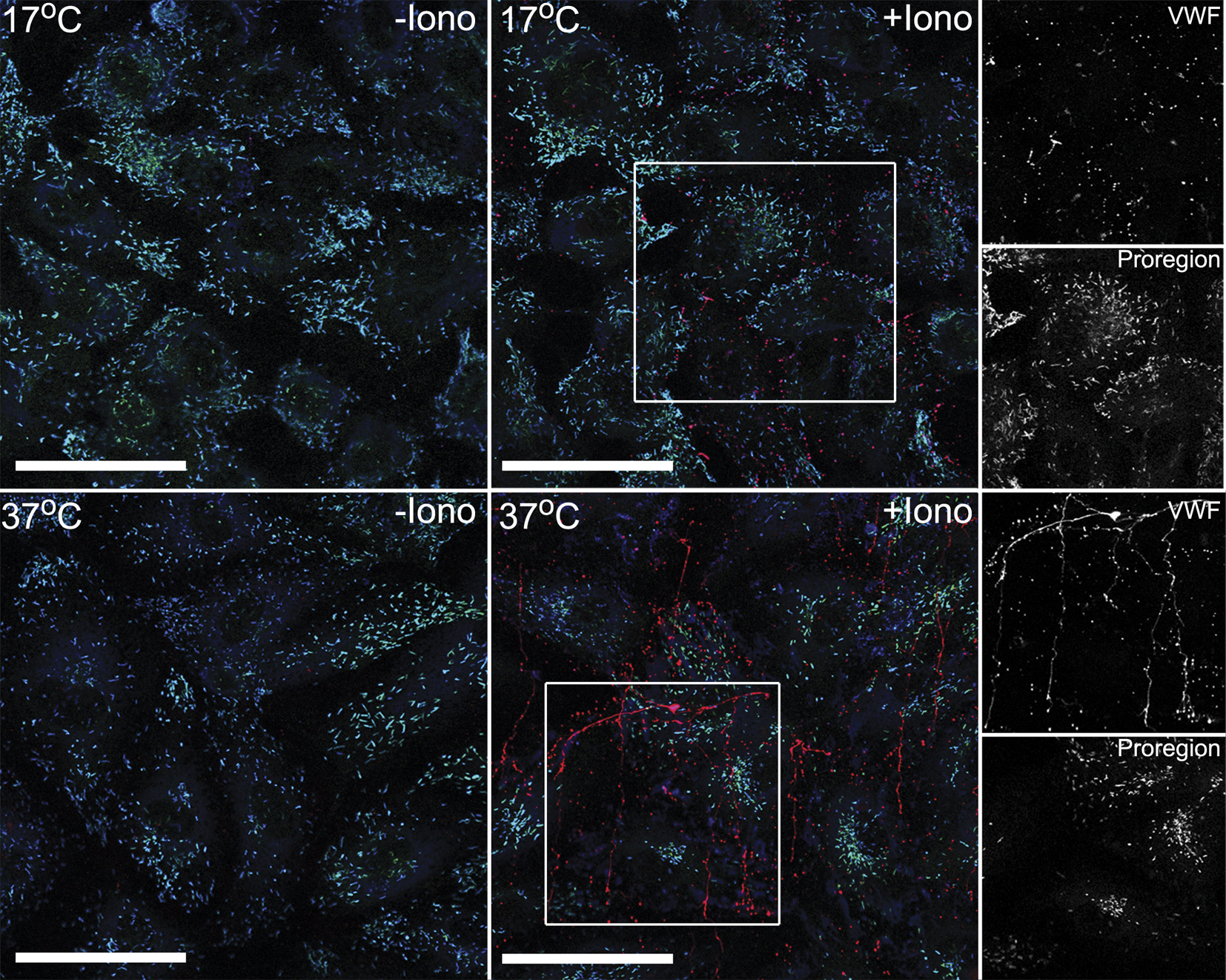

Supplement: Figure S2 — WPB exocytosis at 37°C but not 17°C leads to the formation of long extracellular strings of VWF. Colour merged images of extracellular VWF (red; rabbit anti-human VWF), intracellular VWF (green; sheep anti-human VWF) and proregion (blue; chicken polyclonal antibody specific to the putative C-terminus of human proregion [19] immunoreactivity on control (−Iono) and stimulated (+Iono) cells at the temperatures indicated. Grey scale panels on the right show regions indicated by the white boxes. Images for each antigen were acquired sequentially as single, confocal, optical sections. (Images for the same antigen were acquired using the same confocal settings). Scale bars are 50 µm. (TIF) [file pone.0027314.s002.tif]
